# *ALKBH5* enhances lipid metabolism reprogramming by increasing stability of *FABP5* to promote pancreatic neuroendocrine neoplasms progression in an m6A-*IGF2BP2*-dependent manner

**DOI:** 10.1186/s12967-023-04578-6

**Published:** 2023-10-19

**Authors:** Jinhao Chen, Mujie Ye, Jianan Bai, Zhihui Gong, Lijun Yan, Danyang Gu, Chunhua Hu, Feiyu Lu, Ping Yu, Lin Xu, Yan Wang, Ye Tian, Qiyun Tang

**Affiliations:** 1 Department of Geriatric Gastroenterology, Neuroendocrine Tumor Center, Jiangsu Province Hospital, The First Affiliated Hospital of Nanjing Medical University, Institute of Neuroendocrine Tumor, Nanjing Medical University, No. 300 Guangzhou Road, Nanjing, 210029 China; 2 Digestive Endoscopy, Jiangsu Province Hospital, The First Affiliated Hospital of Nanjing Medical University, Nanjing, China; 3 Department of Gastroenterology, The Friendship Hospital of Ili Kazakh Autonomous Prefecture, Ili & Jiangsu Joint Institute of Health, Yining, 835000 Ili State China

**Keywords:** Pancreatic neuroendocrine neoplasms, *N*6-Methyladenosine (m6A), *ALKBH5*, *FABP5*, Lipid metabolism

## Abstract

The process of post-transcriptional regulation has been recognized to be significantly impacted by the presence of *N*6-methyladenosine (m6A) modification. As an m6A demethylase, *ALKBH5* has been shown to contribute to the progression of different cancers by increasing expression of several oncogenes. Hence, a better understanding of the key targets of *ALKBH5* in cancer cells could potentially lead to the development of new therapeutic targets. However, the specific role of *ALKBH5* in pancreatic neuroendocrine neoplasms (pNENs) remains largely unknown. Here, we demonstrated that *ALKBH5* was up-regulated in pNENs and played a critical role in tumor growth and lipid metabolism. Mechanistically, *ALKBH5* over-expression was found to increase the expression of *FABP5* in an m6A-*IGF2BP2* dependent manner, leading to disorders in lipid metabolism. Additionally, *ALKBH5* was found to activate *PI3K/Akt/mTOR* signaling pathway, resulting in enhanced lipid metabolism and proliferation abilities. In conclusion, our study uncovers the *ALKBH5/IGF2BP2/FABP5/mTOR* axis as a mechanism for aberrant m6A modification in lipid metabolism and highlights a new molecular basis for the development of therapeutic strategies for pNENs treatment.

**Graphical Abstract:**

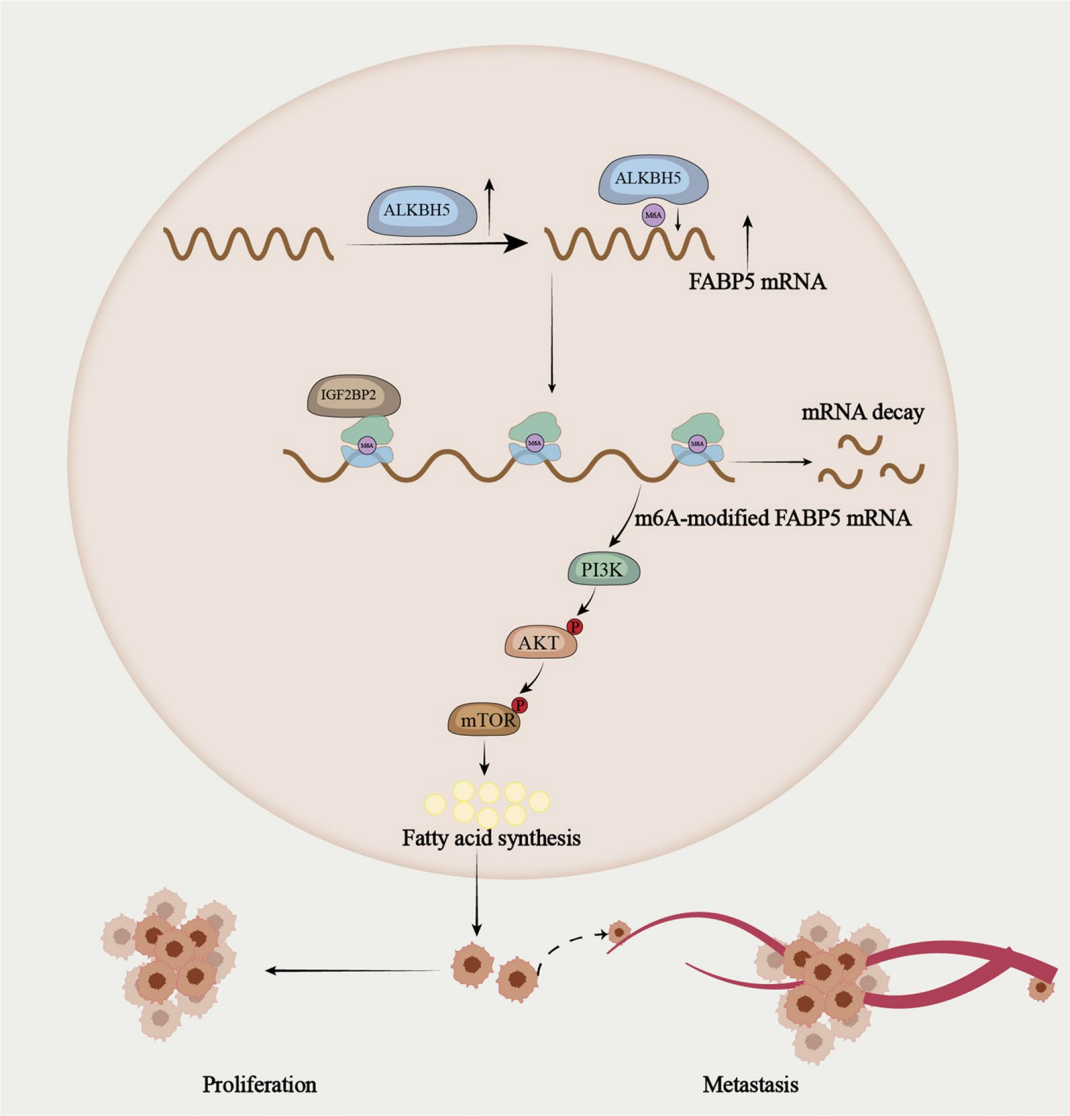

**Supplementary Information:**

The online version contains supplementary material available at 10.1186/s12967-023-04578-6.

## Introduction

Neuroendocrine neoplasms (NENs) originate from the neuroendocrine system and frequently manifest in organs including the lungs, pancreas, and pituitary glands [1]. Nevertheless, the low incidence rates and the inadequacy of experimental models have impeded our comprehension of the mechanisms underlying NEN development [2]. Pancreatic neuroendocrine neoplasms (pNENs) account for 1–2% of pancreatic neoplasms and predominantly arise in the duodenal-pancreatic region, representing the second most frequent pancreatic neoplasm [3]. The prevalence of pancreatic neuroendocrine neoplasms (pNENs), once regarded as uncommon heterogeneous neoplasms, has shown an almost twofold increase to 10% in the last two decades [4, 5]. According to the statistics from SEER, the incidence rate of pNENs exhibited a notable rise of 8.20% between 1996 and 2015. More specifically, the incidence rate of pNET experienced a notable rise of 9.94% among females after 2000, and a significant increase of 7.55% in males following 1993. The upward trend in pNET incidence is expected to persist, with projections indicating that by 2025, the incidence rate will reach 2.23 per 100,000 in females and 2.48 per 100,000 in males [6]. The increasing incidence of pNET could also be partly attributed to a higher number of diagnoses made through imaging studies [7]. As the most frequent primary sites of NENs, surgery is currently the sole potential curative measure for pNENs [8]. However, over half of the patients with pNENs present with locally advanced disease or distant metastasis at the time of diagnosis, resulting in an overall median survival of just 3.6 years [7, 9, 10]. The treatments available for advanced pNENs comprise somatostatin, molecular targeted agents, cytotoxic chemotherapy agents, and immune checkpoint inhibitors [11, 12]. Nonetheless, the current drug regimens for pNENs have demonstrated limited therapeutic efficacy according to past studies [13]. Therefore, research examining the cellular and molecular mechanisms underlying the development of pNENs is urgently required and may pave the way for developing novel therapeutic strategies for patients with advanced diseases.

*N*6-methyladenosine (m6A) is the most abundant RNA base methylation modification among eukaryotes, accounting for 80% of such modifications. Since the discovery of the demethylase *FTO* and *ALKBH5*, m6A has garnered significant attention for its dynamic and reversible nature [14]. The m6A process involves various components, including methyltransferase complexes, also known as “writers”, demethylases or “erasers”, and binding proteins, named “readers” [15]. The main constituents of the methyltransferase complex are Methyltransferase-like 3 (*METTL3*), Methyltransferase-like 14 (*METTL14*), and Wilms’ tumor 1-associated protein (*WTAP*) [16]. Conversely, m6A modification can be eliminated by specific erasers, including *FTO* and *ALKBH5* [17, 18]. Similarly, m6A readers such as *YTHDF1-3* [19], *YTHDC1* [20], Insulin-like growth factor 2 mRNA-binding proteins (*IGF2BPs*) [21], and the heterogeneous nuclear ribonucleoprotein (HNRP) protein family [16], are responsible for recognizing m6A function. Emerging studies have revealed the crucial role of m6A modification played in diverse types of diseases, especially cancers [22, 23]. However, the role of m6A modification in pNENs remains largely unclear.

Metabolic reprogramming is a prevalent hallmark of tumorigenesis, present in many types of cancer, and is considered one of the 14 vital hallmarks [24]. Cancer cells harbor unique metabolic characteristics, such as glucose, lipid, and amino acid metabolism, which facilitate the production of biomass that supports cell duplication and other vital hallmarks of cancer [25]. For glucose metabolism, to support the rapid growth and proliferation of the tumor, cancer cells exhibit heightened glycolysis levels, even when there is excessive oxygen available; this phenomenon is known as the “Warburg effect,” and it is a hallmark of cancer [26]. Additionally, lipid metabolism also plays a crucial role in determining the fate and function of tumor cells, alongside glucose and amino acid metabolism. Although abnormal lipid metabolism in tumor cells has not received as much attention, in recent years, its significance has been increasingly acknowledged. Functioning as lipid transporters, FABPs play an active role in transporting lipids to various cellular components, such as the lipid droplet, endoplasmic reticulum, mitochondria or peroxisome, and nucleus, as well as facilitating autocrine or paracrine signaling outside of the cell [27]. *FABP5*, a petite 15 kDa cytoplasmic protein, mainly participates in the uptake, transport, and metabolism of fatty acids in cell cytoplasm [28]. Present studies have revealed that *FABP5* plays significant roles in different types of cancers, such as lung cancer [29], breast cancer [30], bladder cancer [31], hepatocellular carcinoma [32], clear cell renal cell carcinoma [33], multiple myeloma [34] and so on [35]. However, the role of *FABP5* in pNENs remains unclear and needs to be further explored.

In this work, we demonstrated the high expression and carcinogenesis of *ALKBH5* in pNENs progression in vitro and in vivo. Further studies suggested that *ALKBH5* promoted lipid metabolism by increasing the expression of *FABP5* in an m6A-*IGF2BP2*-dependent manner, which subsequently promoted the malignant behaviors of pNENs. Conversely, the down-regulation of *FABP5* could reverse the increase in lipid metabolism and tumor growth. Overall, our findings identified m6A-modified *FABP5* as a novel metabolic regulator in pNENs development which may be a potential therapeutic target for pNENs treatment.

## Materials and methods

### Cell culture and tissue samples

The human pNENs cell line QGP-1 was obtained from the JCRB cell bank (JCRB0183), which was cultured in RPMI 1640 supplemented with 10% fetal bovine serum (FBS, Yeasen, Shanghai, China), and 1% penicillin–streptomycin. In addition, we also isolated the primary human pNENs cells (we named it PNET) from the pNENs tissues of patients diagnosed with pNENs. The primary human pNENs cell (PNET) were cultured in McCoy’s 5A medium. All cells were cultured in a humidified incubator with 5% CO_2_ at 37 °C. All pNENs tissues and matching adjacent normal tissues were obtained from Jiangsu Province Hospital and were diagnosed as pNENs by Pathology Department of Jiangsu Province Hospital and all consents were signed by every participant.

### Cell proliferation assays

To evaluate the cell proliferation ability, Cell Counting Kit-8 (CCK-8, New Cell & Molecular Biotech), colony formation, and EdU assays were performed. For the CCK-8 assay, 5 × 10^3^ cells were seeded in a 96-well plate with 5 replicates per well and then incubated at 37 °C for 2 h. The absorbance at 450 nm was monitored continuously for 4 days. For the colony formation assay, 2.0 × 10^4^ cells were added to 6-well plates, and the process was repeated thrice. Following 14 days of incubation, the plates were rinsed twice with phosphate-buffered saline (PBS), fixed with paraformaldehyde for 15 min, and stained with 0.1% crystal violet solution for 15 min for subsequent analysis. As for the EdU assay, cells in a 96-well plate were subjected to 50 μM EdU for 2 h and then stained according to the instructions provided (RiboBio, Guangzhou, China).

### Cell migration and invasion assays

To determine cell migration and invasion capacity, we employed 24-well cell culture plates with 8-μm micropore inserts. For cell migration assays, 1 × 10^5^ cells in serum-free medium were placed in the upper chamber and incubated for 48 h. For cell invasion assays, 2 × 10^5^ cells were seeded in the upper chamber with 50 μL of Matrigel (Becton, Dickinson) and incubated for 48 h. In both cases, the lower chamber was filled with a conditioned culture medium containing 30% FBS. After 48 h, cells that had invaded the bottom were fixed with 4% paraformaldehyde and stained with 0.25% crystal violet solution for 30 min.

### Assays of lipid metabolism

To quantify the lipid droplets in cells, we performed Nile red staining. pNENs cells that were transfected were seeded in 96 well plates. Once the cells had attained confluency between 60 and 80%, they were fixed in 4% paraformaldehyde for 15 min. Afterward, the cells were incubated with Nile Red working fluid for 20 min, followed by staining with DAPI (Beyotime, Nantong, China) for 20 min at room temperature. The fluorescence intensity imaging of Nile Red and DAPI was acquired using fluorescence microscopy. The fluorescence intensities were quantified using ImageJ software. Quantification of fatty acids (FAs) was performed by using a CheKine™ Micro Free Fat Acid (FFA) Assay Kit (abbkine). Triglyceride and cholesterol contents were performed using EnzyChrom triglyceride and cholesterol kits (Bioassay Systems). All assays were performed following the manufacturer’s instructions.

### Quantitative real time-polymerase chain reaction (qRT-PCR) and RNA-seq

Total RNA from cells was isolated using the trizol reagent (Vazyme, Nanjing, China). RNA was quantified using a Nanodrop 2000. Subsequently, 5 µg of RNA was used for reverse transcription with the PrimeScript RT Reagent Kit with gDNA Eraser (Yeasen, Shanghai, China). Real-time PCR was then conducted according to the manufacturer’s instructions using ChamQ Universal SYBR qPCR Master Mix. For RNA-seq, RNA samples were sequenced by Lianchuan Biotech (Hangzhou, China) and analyzed using the OmicStudio tools at https://www.omicstudio.cn/tool.The primers utilized are specified in Additional file 1: Table S1.

### Protein extraction and western blot analysis

Total proteins from cultured cells were lysed with cold NP40 lysis buffer (Beyotime) containing a protease inhibitor cocktail (Roche, Mannheim, Germany). The protein concentration was determined using a BCA Protein Assay Kit (Beyotime). Samples were separated on an 10% gel by SDS-PAGE. Nitrocellulose membranes were blocked with a blocking buffer and incubated with the appropriate primary antibody. The membranes were then blocked with 8% skim milk in TBST, incubated with primary antibodies (listed in Additional file 1: Table S3) overnight at 4 °C, and then incubated with HRP-conjugated secondary antibodies for one hour at room temperature. The blots were imaged with Immobilon™ Western Chemiluminescent HRP Substrate (Millipore) and the ChemiDoc™ XRS + imaging system (Bio-Rad).

### Plasmid construction

To enable *ALKBH5* and *FABP5* over-expression, we amplified and cloned the cDNA encoding the *ALKBH5* and *FABP5* CDS region into the pCDH-CMV-MCS-EF1-Puro lentivirus vector. Similarly, we used recombinant lentiviruses containing sh-*ALKBH5*, sh-*IGF2BP2*, or *FABP5* in the PLKO1 vector, to construct *ALKBH5*, *IGF2BP2*, and *FABP5* knockdown stable cell lines (listed in Additional file 1: Table S2). These plasmids were from Genomeditech (Shanghai, China). We accomplished plasmids transfection in 293 T cells using PEI (Polysciences, USA) with serum-free medium, followed by the addition of the corresponding serum after 6 h. Subsequently, we harvested the viral supernatant through a 0.45-μm filter after 48 h and applied it to cells having 50% confluence. Treatment with 2 μg/ml puromycin for 7 days was used to select stable cell lines.

### Immunofluorescence

Cells were planted in 96 well plates overnight to reach confluency between 60 and 80%. Next, cells were washed with PBS three times and then fixed with 4% paraformaldehyde for 15 min. Then, cells were incubated with 0.2% Triton X-100 for 15 min and blocked in 3% BSA for 30 min. After three times washing of PBST, the cells were incubated at 4° overnight with primary antibody against *ALKBH5* and *FABP5*. Then cells were washed by PBST three times and subsequent 1-h incubation at room temperature with secondary antibodies and DAPI. The fluorescence intensity imaging was acquired using fluorescence microscopy.

### RNA stability assay

Cells were treated with actinomycin D (MCE, China) at a final concentration of 5 μg/mL for the indicated time periods and collected. Total RNAs were extracted and analyzed with qRT-PCR.

### Quantification of global *N*6-methyladenosine levels

To determine the global level of RNA N6-methyladenosine (m6A), we employed the EpiQuik m6A RNA Methylation Kit (Epigentek, USA) in our study. Initially, we isolated total RNA from cells or tissues utilizing TRIzol reagent and proceeded to bind the RNA onto strip wells using the RNA high-binding solution. Subsequently, we added capture and detection antibodies in a sequential manner into each well. Finally, we compared and measured the absorbance value at a wavelength of 450 nm to ascertain the relative m6A level.

### Methylated RNA immunoprecipitation sequencing (MeRIP-seq)

The target gene was selected by MeRIP using MeRIP m6A Kit (Merck Millipore) following the provider’s requirements. Specially speaking, total RNAs were extracted from *ALKBH5* knockdown or empty vector QGP-1 cells using Seq-Star Poly(A) mRNA Isolation Kit. Next, the RNA was fragmented and incubated with m6A antibody to deposit *FABP5*. After the concentration of m6A mRNA fragment and construction of the RNA-seq library for sequencing on the Illumina HiSeq 4000 platform. Subsequently, the abundance of *FABP5* was tested by qRT-PCR and normalized to the input mRNAs.

### RNA immunoprecipitation (RIP) assays

In accordance with the manufacturer’s instructions, the Magna RIP Kit (17–700, Millipore, MA) was utilized to perform the RIP assay. Specifically, 5 μg of anti-*ALKBH5* (Abcam, USA), anti-*IGF2BP2* (Abcam, USA), or anti-*N*6-methyladenosine (m6A) (Abcam, USA) and anti-rabbit IgG (Millipore, Germany) were incubated with 40 μL magnetic beads, prior to the addition of cell lysates (approximately 5 × 10^7^ cells per sample). Next, the RNA–protein IP complexes were washed six times. Following treatment with proteinase K, the RNAs of interest were extracted and purified from the immuno precipitated complex for further qRT-PCR analysis. The relative enrichment was normalized with the input.

### Mouse xenograft model

For tumor xenograft models, QGP-1cells (5 × 10^6^) with *ALKBH5* over-expression, *ALKBH5* over-expression with *FABP5* knockdown, and negative control were subcutaneously injected into the right axilla of female BALB/c nude mice (4–6 weeks). After 4 weeks, the mice were sacrificed via a form of euthanasia. The tumors were weighed, imaged, and fixed in 4% paraformaldehyde or frozen for further analysis. Tumor volume was measured by the following formula: volume = length × width^2^ × 1/2. All animal experiments were approved by the Institutional Animal Care and Use Committee (IACUC) of Nanjing Medical University.

### Statistical analysis

Statistics were analyzed using GraphPad Prism 8.0 (GraphPad, Inc., USA). Comparisons between different groups were calculated using Student’s t-test. Experiments were independently repeated at least three times. Representative data was exhibited as the means ± SD. p-values for every result were labeled on figures, and p < 0.05 was reckoned as statistically significant.

## Results

### *ALKBH5* is increased in pancreatic neuroendocrine neoplasms

**Fig. 1:**
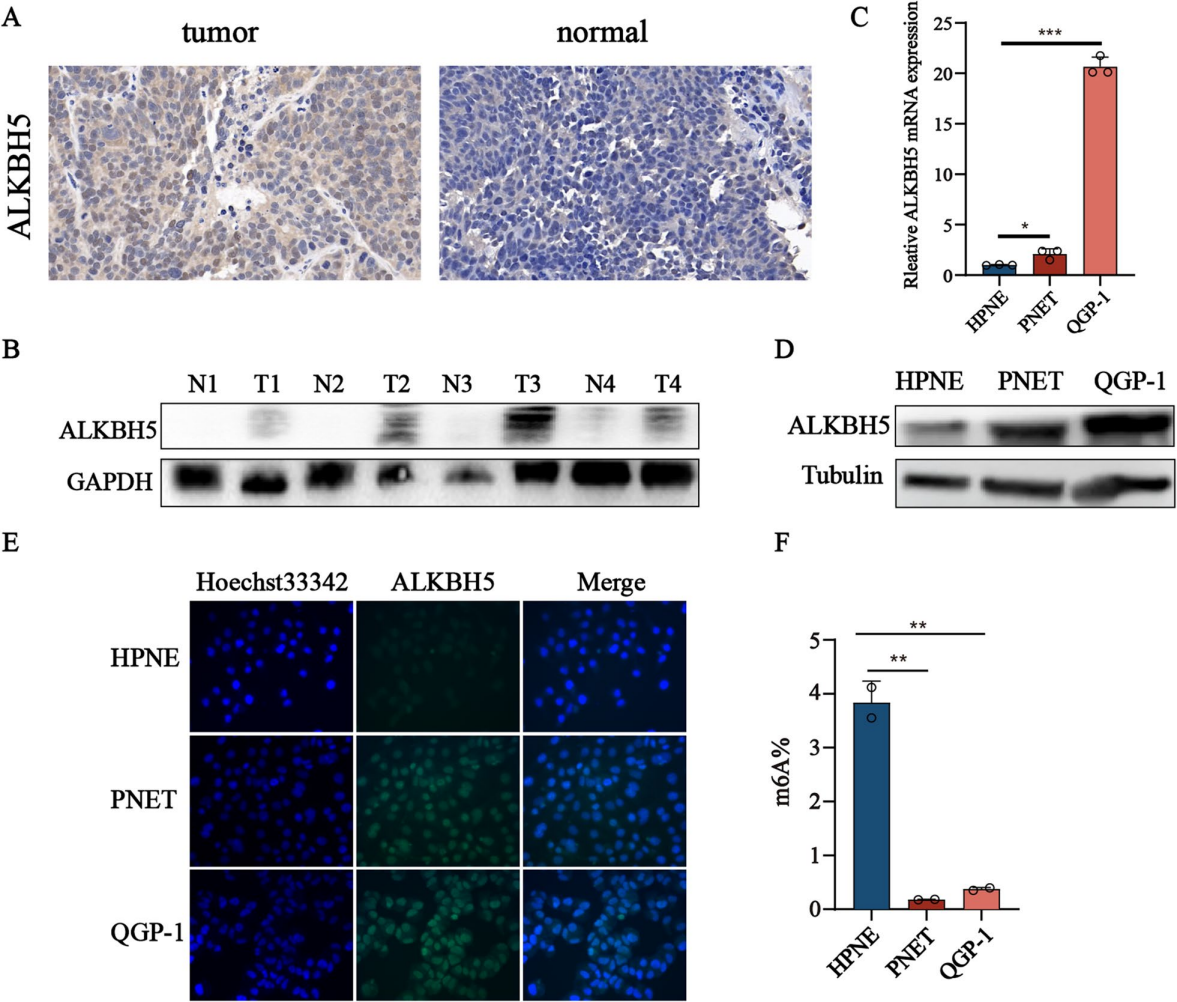
Increased expression of *ALKBH5* is observed in pNENs. **A** Representative images of *ALKBH5* immunohistochemistry staining in pNENs samples were shown. **B** The protein expression of *ALKBH5* from four pairs of pNENs samples showed by western blots (T: tumor, N: normal). **C**, **D** The mRNA and protein expression of *ALKBH5* in normal pancreatic cell lines compared to pNENs. **E** The expression of *ALKBH5* was detected by immunofluorescent imaging. **F** The amount of m6A in normal pancreatic cell lines HPNE and pNENs cell lines PENT and QGP-1. **p < 0.01, ***p < 0.001

To investigate the potential role of *ALKBH5* in pNENs, we initially examined the expression of *ALKBH5* in tumor tissues and normal tissues by using an immunohistochemistry (Fig. 1A). Subsequently, we analyzed the expression of *ALKBH5* protein in four pairs of pNENs and found that the most of them exhibited significantly higher expression levels than the corresponding normal adjacent tissues (Fig. 1B). Consistent with these results of tissues, qRT-PCR and western blot analyses revealed that pNENs cell lines also displayed elevated levels of *ALKBH5* mRNA and protein expression compared to normal pancreatic cells (Fig. 1C, D). Furthermore, the results of the immunofluorescence assay indicated the altered expression of *ALKBH5* in pNENs cell lines compared to the normal pancreatic cells (Fig. 1E). To investigate the potential involvement of m6A modification in pNENs, we also compared the global m6A levels in pNENs cell lines to those of normal pancreatic cells. The findings suggest that the overall levels of global m6A expression were significantly lower in pNENs compared to the normal pancreatic cells (Fig. 1F). Finally, we also examined the protein expression of some common m6A regulators (such as *FTO*, *ALKBH5*, *METTL3*, *WTAP*, *METTL14*, and *YTHDC1*) which indicated the role of the m6A modification in the progression in pNENs (Additional file 1: Figure S1A). The results showed the m6A writers highly expressed while m6A erasers showed lower expression which indicated the high m6A modification in pNENs. Taken together, these results highlight the possible indispensable role of *ALKBH5* in the development and progression of pNENs.

### *ALKBH5* promotes the malignant progression of pNENs in vitro and in vivo

**Fig. 2:**
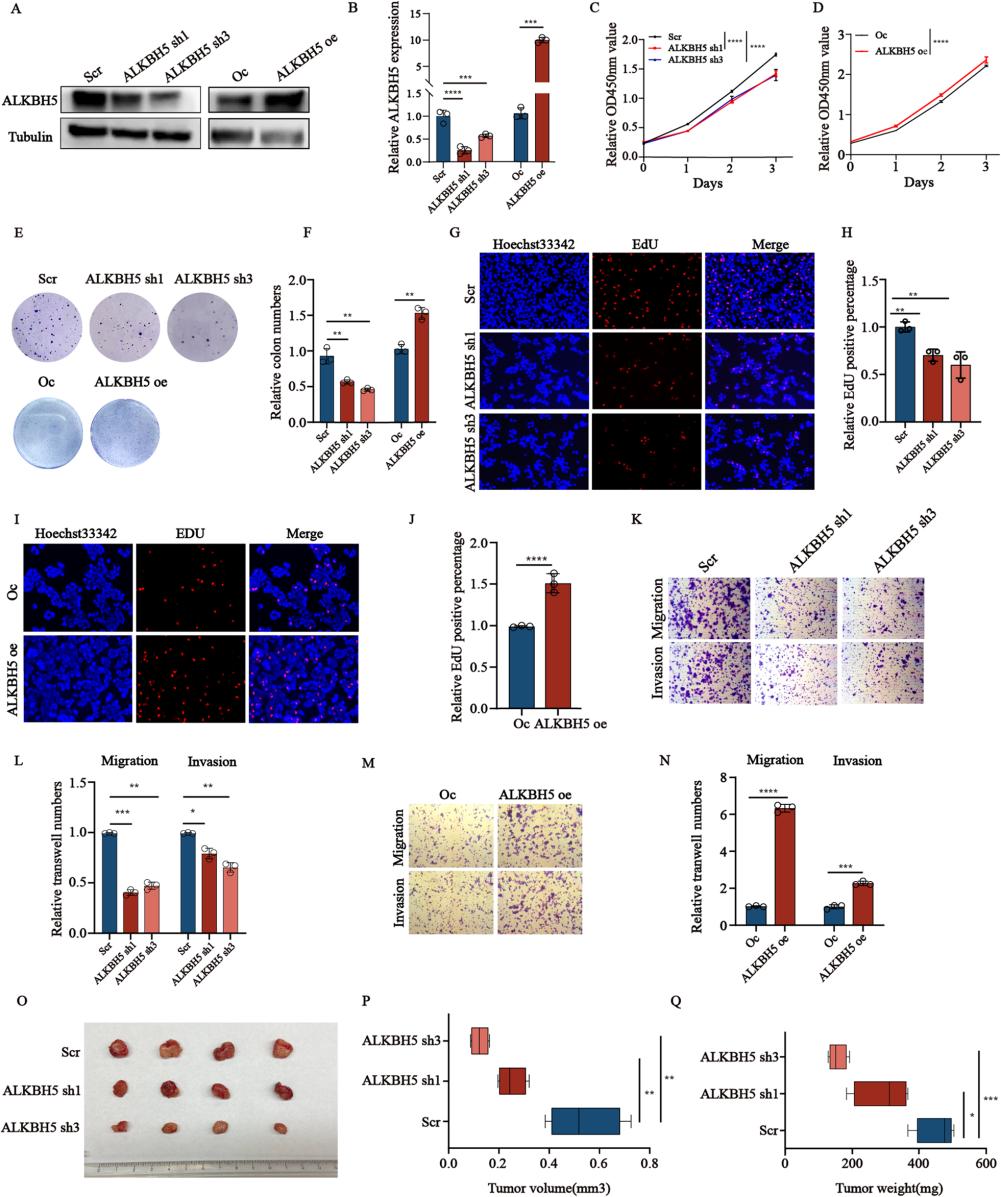
*ALKBH5* knockdown inhibits proliferation, migration, and invasion of QGP-1 cells in vitro and in vivo. **A**, **B** The mRNA and protein expression of *ALKBH5 in* QGP-1 cells were assessed by qRT-PCR and western blotting (Scr represents scramble which means control group with disrupted rna sequence; Oc represents overexpression control). **C**, **D** CCK-8 proliferation assays were carried out in QGP-1 cells with *ALKBH5* knockdown and overexpression. **E**, **F** Colony formation assays were conducted in QGP-1 cells with *ALKBH5* knockdown and overexpression. Column diagrams showed the relative colony numbers of each group. **G**–**J** EdU assays were carried out to evaluate the proliferation of cells with *ALKBH5* knockdown and overexpression and a positive rate of EdU was calculated, magnification: ×200. **K**–**N** Representative images of transwell assays to evaluate the migration and invasion capacity of QGP-1 cells with *ALKBH5* knockdown and overexpression, magnification: ×100. Quantification data showed the relative transwell numbers of cells which passed through the chamber membrane. **O**–**Q** Representative images of tumors and comparison of the tumor volume and weight between *ALKBH5*-deficient groups and scramble groups in QGP-1 cells. *p < 0.01, **p < 0.01, ***p < 0.001

To explore the potential promoting effects of *ALKBH5* on tumor development, lentiviral transfection technology was utilized to construct the stabled transfected cell lines with *ALKBH5* knockdown and ALKBH5 overexpression in QGP-1 cells (Fig. 2A, B). *ALKBH5* inhibition led to a significant reduction in proliferation and colony formation in pNENs cells, as indicated by the results of the CCK8 assay and colon formation, while *ALKBH5* overexpression resulted in the opposite effects. (Fig. 2C–F). Furthermore, the EdU assay also revealed that DNA replication activity in QGP-1 cells could be reduced in *ALKBH5*-deficient cells and enhanced in *ALKBH5*-overexpression cells (Fig. 2G–J). Additionally, the migration and invasion ability of QGP-1 cells were also impaired by *ALKBH5* depletion and *ALKBH5* overexpression (Fig. 2K–N). These results suggested that *ALKBH5* serves as an oncogene driving pNENs development.

Moreover, the stable transfected cell lines of *ALKBH5* knockdown and overexpression in PNET were also constructed which showed significantly down-regulated and up-regulated *ALKBH5* mRNA and protein levels (Additional file 1: Figure S1B, C). The CCK8 assay and colon formation assay both showed that *ALKBH5* knockdown and over-expression in PNET caused significant inhibition and enhancement of cell viability and colony forming ability (Additional file 1: Figure S1D–G). The result of the EdU assay also showed that *ALKBH5* knockdown and overexpression had the opposite effects on the DNA replication activity in PNET cells (Additional file 1: Figure S1H–K). Finally, the transwell assay also revealed that *ALKBH5* knockdown inhibited the migration and invasion ability of PNET cells shown by the amount of the stained cells at the bottom of the chamber while *ALKBH5* overexpression showed the opposite results (Additional file 1: Figure S1L–O). To explore the function of *ALKBH5* in vivo, we construct the tumor xenograft models using QGP-1 cells with *ALKBH5* knockdown. The results revealed that *ALKBH5* knockdown inhibited tumor growth and weight (Fig. 1O–Q). The above results indicate that *ALKBH5* plays a crucial role in tumor development.

### *ALKBH5* regulates fatty metabolism pathways

**Fig. 3:**
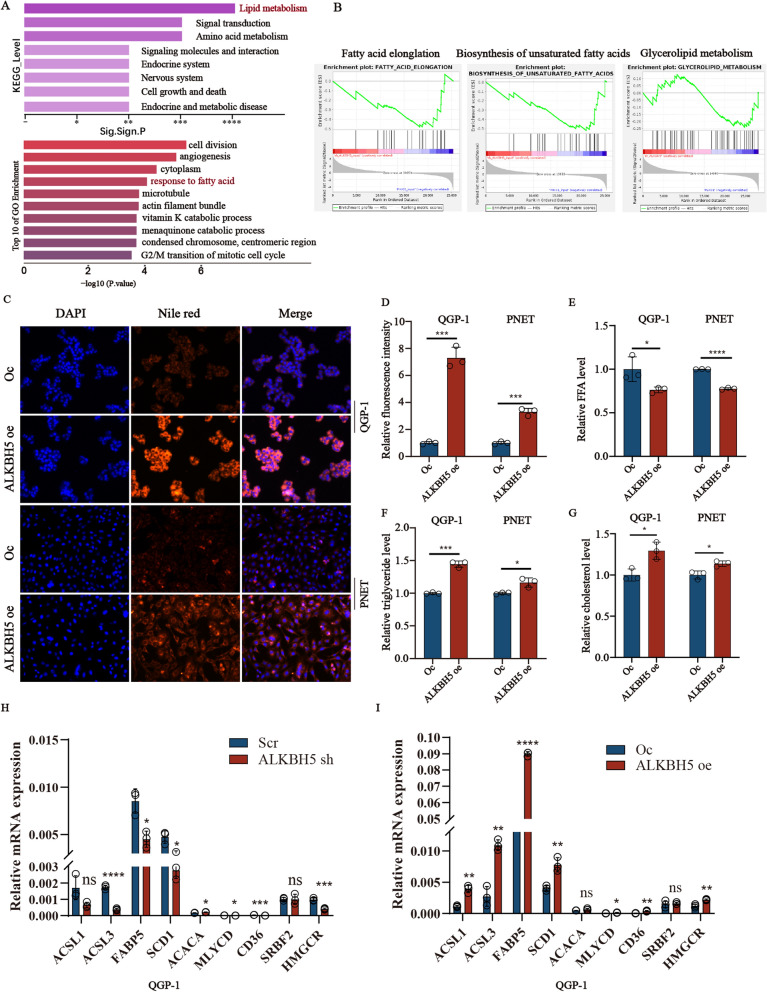
*ALKBH5* regulates lipid metabolism. **A** Enrichment analysis of KEEG and GO signal pathways. **B** Individual GSEA plots of fatty acid elongation, glycerolipid metabolism, and biosynthesis of unsaturated acid pathway in RNA-seq data from QGP-1 cells with *ALKBH5* knockdown. **C**, **D** LDs (lipid droplets) were detected using Nile red in indicated cells with *ALKBH5* over-expression, magnification: ×200, along with the results of relative fluorescence intensity. **E**–**G** The relative amounts of free fatty acids, triglycerides, and cholesterol were measured in cells with *ALKBH5* over-expression. **H**, **I** The mRNA expression of dysregulation of genes involved in fatty acid synthesis (*ACSL1, ACSL3, FABP5, SCD1, ACACA, MLYCD*), fatty acid uptake (*CD36*), cholesterol biosynthesis (*SREBF2* and *HMGCR*) in QGP-1 cells with *ALKBH5* knockdown and over-expression. *p < 0.01, **p < 0.01, ***p < 0.001

To determine potential *ALKBH5*-regulated signaling pathways, we initially performed RNA-seq between *ALKBH5* depletion cells and control cells. The signal about tumor growth, including cell growth and death, cell division, angiogenesis, and G2/M transition of the mitotic cell cycle were significantly enriched, and correspond to the previous results of cell phenotype. The classical *MAPK* signaling pathway and *PI3K–Akt–mTOR* signaling pathway were expectedly enriched. Interestingly, two lipid metabolism-related pathways (lipid metabolism and response to fatty acid) were also identified by KEGG analysis and GO enrichment respectively (Fig. 3A). As mentioned before, dysregulated lipid metabolism plays an indispensable role in many cancers. However, the role of lipid metabolism in pNENs influenced by *ALKBH5* has not been explored. Therefore, we next focused on the specific mechanism of *ALKBH5* on lipid metabolism in pNENs. We firstly observed the biosynthesis of unsaturated fatty acid, fatty acid elongation, and glycerolipid metabolism pathways were significantly downregulated in *ALKBH5*-knockdown cells revealed by GSEA analysis (Fig. 3B). Moreover, we also detected the difference in the related index of lipid metabolism. The results revealed that *ALKBH5* over-expression increased the total lipid droplet levels (Fig. 3C, D), suggesting increased lipid storage. *ALKBH5* over-expression also decreased the content of free fatty acid which showed the activity of lipolysis may be enhanced by *ALKBH5* (Fig. 3E). The amounts of total cholesterol and triglycerides also increased in cells with *ALKBH5* over-expression (Fig. 3F, G). In addition, abnormal lipid metabolism could be induced by the enhancement of lipid biosynthesis, decreased lipid catabolism, and increased fatty acid uptake. Hence, we also analyzed the expression of key molecules in QGP-1 cells with *ALKBH5* knockdown and overexpression involved in lipid metabolism (Fig. 3H, I), including fatty acid synthesis (*ACSL1, ACSL3, FABP5, SCD1, ACACA, MLYCD*), fatty acid uptake (*CD36*), cholesterol biosynthesis (*SRBF2, HMGCR*). The mRNA expression results revealed that the majority of molecules related to fatty acid synthesis, especially for *FABP5*, and fatty acid uptake were promoted by *ALKBH5*. However, the molecules related to cholesterol biosynthesis seem to be weakly influenced by *ALKBH5*. Taken together, the above results all showed that *ALKBH5* may play a crucial role in regulating lipid metabolism for the development of pNENs.

### *FABP5 *has been identified as the target gene regulated by *ALKBH5*

**Fig. 4:**
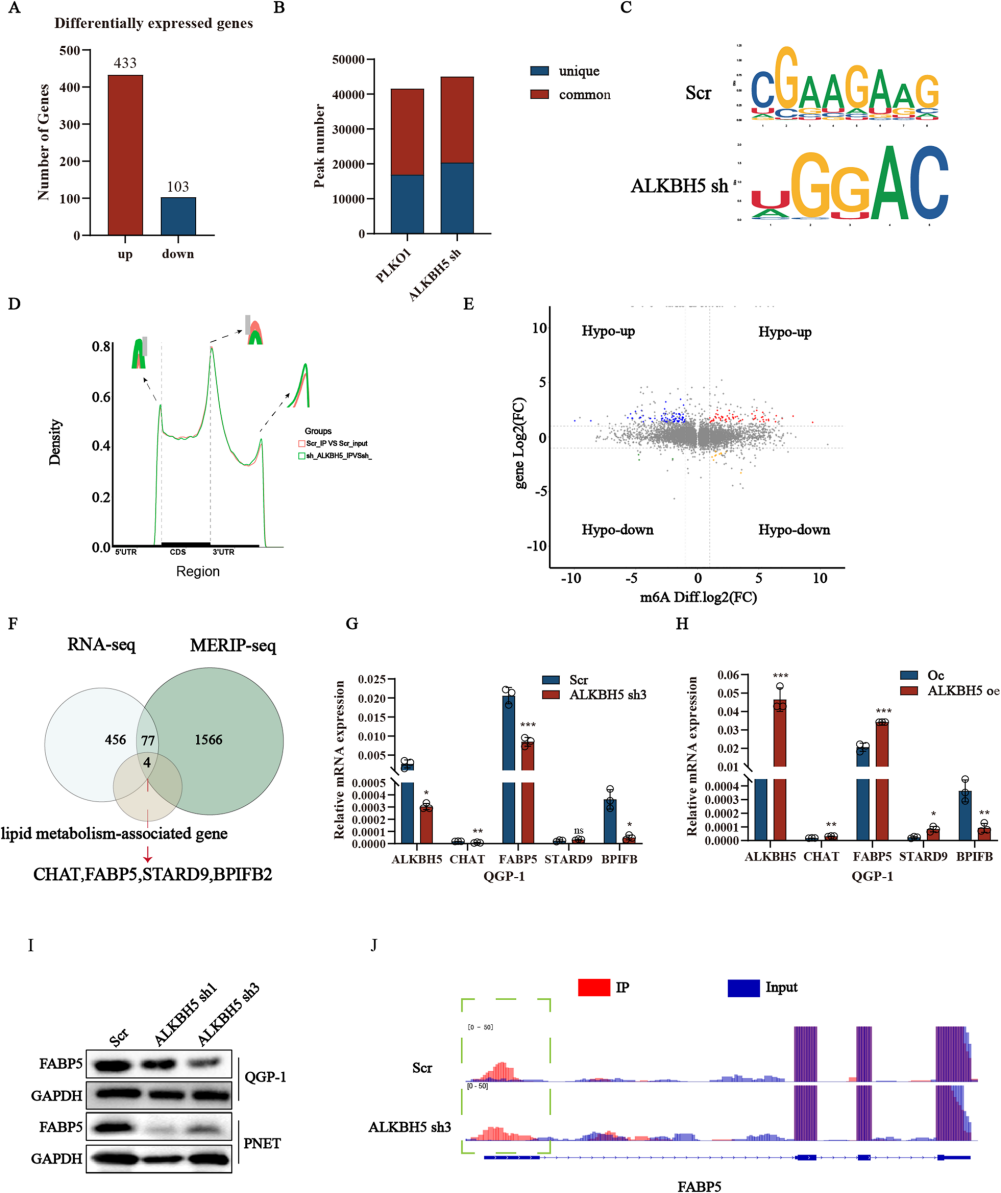
*FABP5* is a functionally important target gene of *ALKBH5*. **A** Differentially expressed genes between *ALKBH5* knockdown and control groups in QGP-1 cells as determined by RNA-sequencing. **B** Peak profiles in m6A modification after *ALKBH5* knockdown in QGP-1 cells as shown by MeRIP-sequencing. **C** The m6A consensus motif is present in QGP-1 cells. **D** Distribution of m6A peaks across the length of mRNAs in QGP-1 cells with or without *ALKBH5* knockdown. **E** The volcano plot showed the distribution of genes both differential (up or down) methylation level and differential (up or down) gene expression level in *ALKBH5* knockdown and control groups. **F** Venn diagram showed the down-regulated genes, genes with elevated m6A methylation levels, and lipid-associated genes after *ALKBH5* knockdown. **G**, **H** The validation of four candidate genes was verified by qRT-PCR in QGP-1 cells. **I** The protein expression of *FABP5* in cells with *ALKBH5* knockdown. **J** The relative abundance of m6A sites along *FABP5* mRNA in QGP-1 cells with or without *ALKBH5* knockdown, as detected by MeRIP-seq. *p < 0.01, **p < 0.01, ***p < 0.001

To further insight into the mechanism of *ALKBH5* regulating lipid metabolism in gene expression, both transcriptome and epitranscriptome sequencing in the QGP-1 cells inhibiting *ALKBH5* and the vector group were performed. For the transcriptome sequencing data, a total of 537 genes was identified with significant differences and P-value was less than 0.05, including 433 up-regulated and 103 down-regulated genes (Fig. 4A). For the meRIP-seq date, we identified 16,950 unique m6A peaks in control group, 20,395 unique m6A peaks in sh-*ALKBH5* group and 24,632 shared m6A peaks in both groups (Fig. 4B). Furthermore, the m6A modification peaks were mainly enriched in the intron region of the genes in both groups, and the UGGAC was the most common consensus motif in pNENs with *ALKBH5* knockdown (Fig. 4C). ALKBH5 knockdown increased m6A enrichment primary in the 3ʹUTR and 5ʹUTR region (Fig. 4D). Next, we combined Methylated RNA immunoprecipitation (MeRIP) with an m6A-specific antibody followed by RNA sequencing (MeRIP-seq) and RNA sequencing to accurately identify the downstream targets of *ALKBH5* (Fig. 4E). According to the overlapping part of RNA-seq and MERIP-seq between control and *ALKBH5* knockdown, we found 201 genes with significant differences. Among this, we also eliminated 113 genes with down-regulated m6a levels which were inconsistent with high global m6A levels regulated by *ALKBH5* knockdown. Moreover, the remaining four genes involved in lipid metabolism were singled out, including *CHAT, FABP5, STARD9*, and *BPIFB2* (Fig. 4F). We next verified the mRNA levels of these candidate genes. The results showed *FABP5* was the gene most significantly changed examined by qRT-PCR in *ALKBH5* knockdown and over-expression cells (Fig. 4G, H). We also verified the protein expression of *FABP5* in *ALKBH5* knockdown cell lines (Fig. 4I). Furthermore, we predicted the m6A modification sites using IGV analysis. The results indicated that the peak in cells with *ALKBH5* knockdown mainly localized in the 5ʹ untranslated region (UTR) (Fig. 4J), which is in line with previous studies suggesting that m6A modification in the 5ʹ UTR is closely associated with energy metabolism. The above results all showed *FABP5* seems the target gene regulated by *ALKBH5* in the development of cancer lipid metabolism.

### *ALKBH5* over-expression up-regulated *FABP5* mRNA levels in an m6A-*IGF2BP2*-dependent manner

**Fig. 5:**
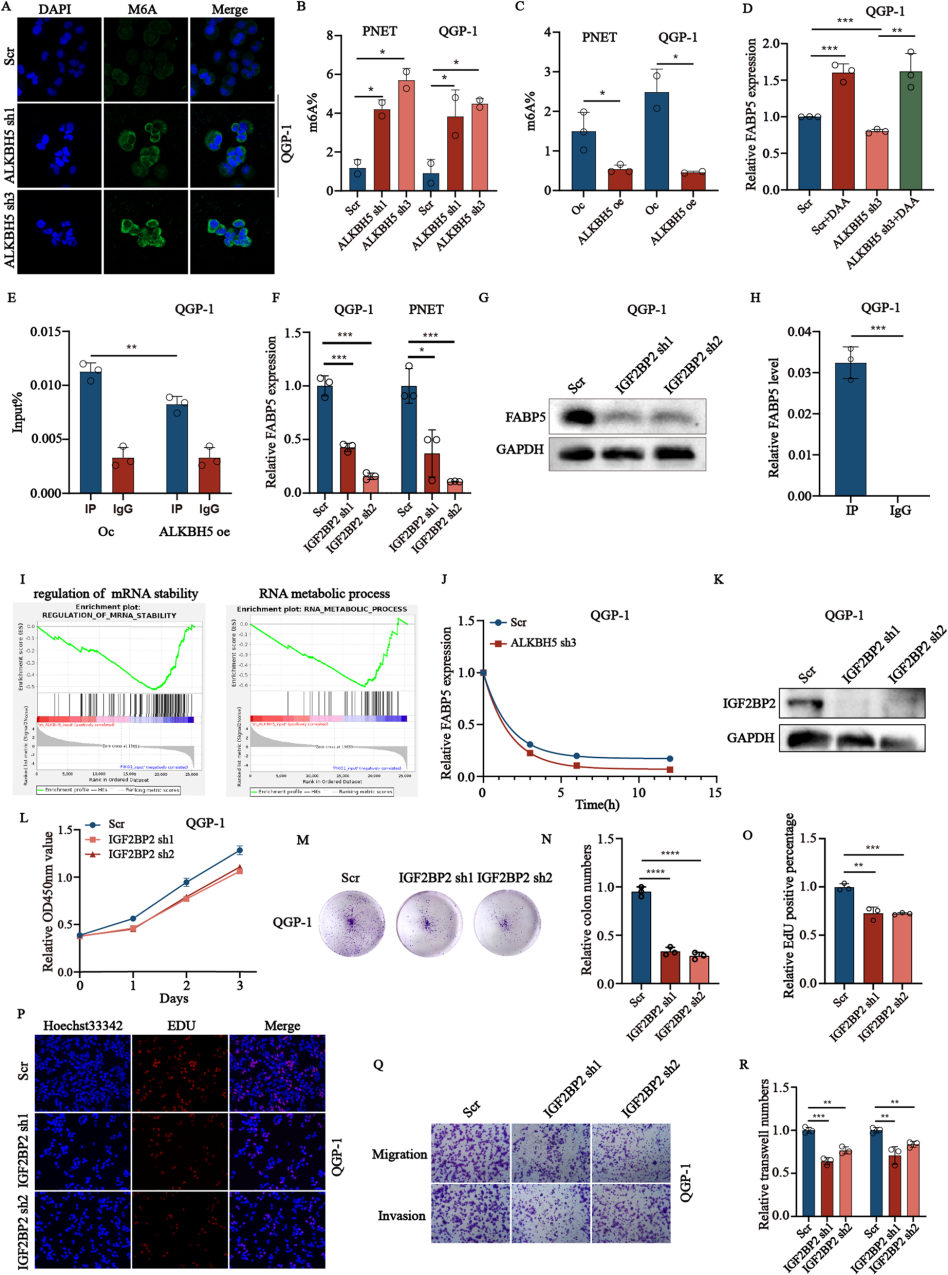
*ALKBH5* stabilizes *FABP5* mRNA in an m6A-dependent manner. **A** The expression of m6A in QGP-1 cells with *ALKBH5* knockdown was detected by immunofluorescent imaging. **B**, **C** The global m6A levels in *ALKBH5* knockdown and overexpression cell lines. **D** The relative expression of *FABP5* mRNA in QGP-1 cells treated with DAA. **E** RIP-qRT-PCR revealing binding enrichment of m6A to *FABP5* mRNA in QGP-1 cells with or without *ALKBH5* knockdown. **F** The relative *FABP5* mRNA expression in cells with *IGF2BP2* knockdown. **G** The protein expression of *FABP5* in QGP-1 cells with *IGF2BP2* knockdown was verified by western blots. **H** RIP-qRT-PCR revealing binding enrichment of *IGF2BP2* to *FABP5* in QGP-1 cells. **I** Individual GSEA plots of regulation of mRNA metabolic process and regulation of mRNA stability pathway in RNA-seq data from QGP-1 cells with *ALKBH5* knockdown. **J** *FABP5* mRNA half-life (t1/2) was tested at the indicated time points by qRT-PCR in QGP-1 cells with *ALKBH5* knockdown. **K** The knockdown rate of *IGF2BP2* was verified by western blots. *IG2BP2* inhibition inhibits the growth and motility of pNENs. The CCK-8 (**L**), colony formation (**M**, **N**), and EdU (**O**, **P**) assays were applied to evaluate the proliferation ability of QGP-1 cells with the knockdown of *IGF2BP2*. **Q**, **R** Transwell assays of QGP-1 cells with *IGF2BP2* knockdown were applied to measure their migration and invasion abilities, magnification: × 100. *p < 0.01, **p < 0.01, ***p < 0.001

Next, to further demonstrated whether *ALKBH5* regulates *FABP5* in an m6A-dependant manner, we first performed the immunofluorescence to examine the expression of the m6A levels in QGP-1 cells with *ALKBH5* knockdown (Fig. 5A). Next, we also examined the global m6A levels in *ALKBH5* knockdown and over-expression cell lines. The results showed that the m6A levels were also changed in corresponding cell lines (Fig. 5B, C). We also treated QGP-1cells with DAA, an RNA methylation inhibitor, to evaluate the expression of *FABP5* in *ALKBH5* knockdown cell lines. The result also showed the amount of m6A and *FABP5* was negatively correlated (Fig. 5D). In addition, we conducted RIP to investigate the close interaction between m6A and *FABP5*. It revealed that the successful combination between m6a and *FABP5* and the less combination in *ALKBH5* over-expression cells (Fig. 5E). Next, we also examined the specific readers when *FABP5* was regulated by *ALKBH5*. *IGF2BP2* was predicated to bind and recognized these m6A modifications by the software prediction and verified by qRT-PCR and western blots (Fig. 5F, G). RIP results also indicated that *IGF2BP2* directly binds *FABP5* (Fig. 5H). The above results all revealed that *IGF2BP2* interacted with *FABP5* through m6A modification. Moreover, it has been demonstrated *ALKBH5* plays an indispensable role in mRNA stability [36]. From the sequencing of me-RIP, we found the m6A modification of *FABP5* was located in 5ʹUTR which may participate in mRNA stability. GSEA results based on RNA-seq data showed that the RNA metabolic process and regulation of mRNA stability signaling pathway was inhibited in the *ALKBH5* knockdown group compared to the control group (Fig. 5I). By treating cells with ActD to test this hypothesis, which inhibits nascent mRNA transcription, we detected the remaining intracellular mRNA in QGP-1cells with or without *ALKBH5* knockdown. We found that the expression of remaining mRNA levels of *FABP5* deceased much faster compared with control cells, suggesting that *ALKBH5* sustained the mRNA stability of *FABP5* (Fig. 5J). To further explore the role of *IGF2BP2* functions in pNENs, we also knockdown the expression of *IGF2BP2* in QGP-1 cells (Fig. 5K) and performed a series of phenotypic experiments in *IGF2BP2* deficient cells which revealed the carcinogenic effect of *ALKBH5* played in pNENs (Fig. 5L–R).

### *FABP5* restores the malignant effects of *ALKBH5* in pNENs in vitro and in vivo

**Fig. 6:**
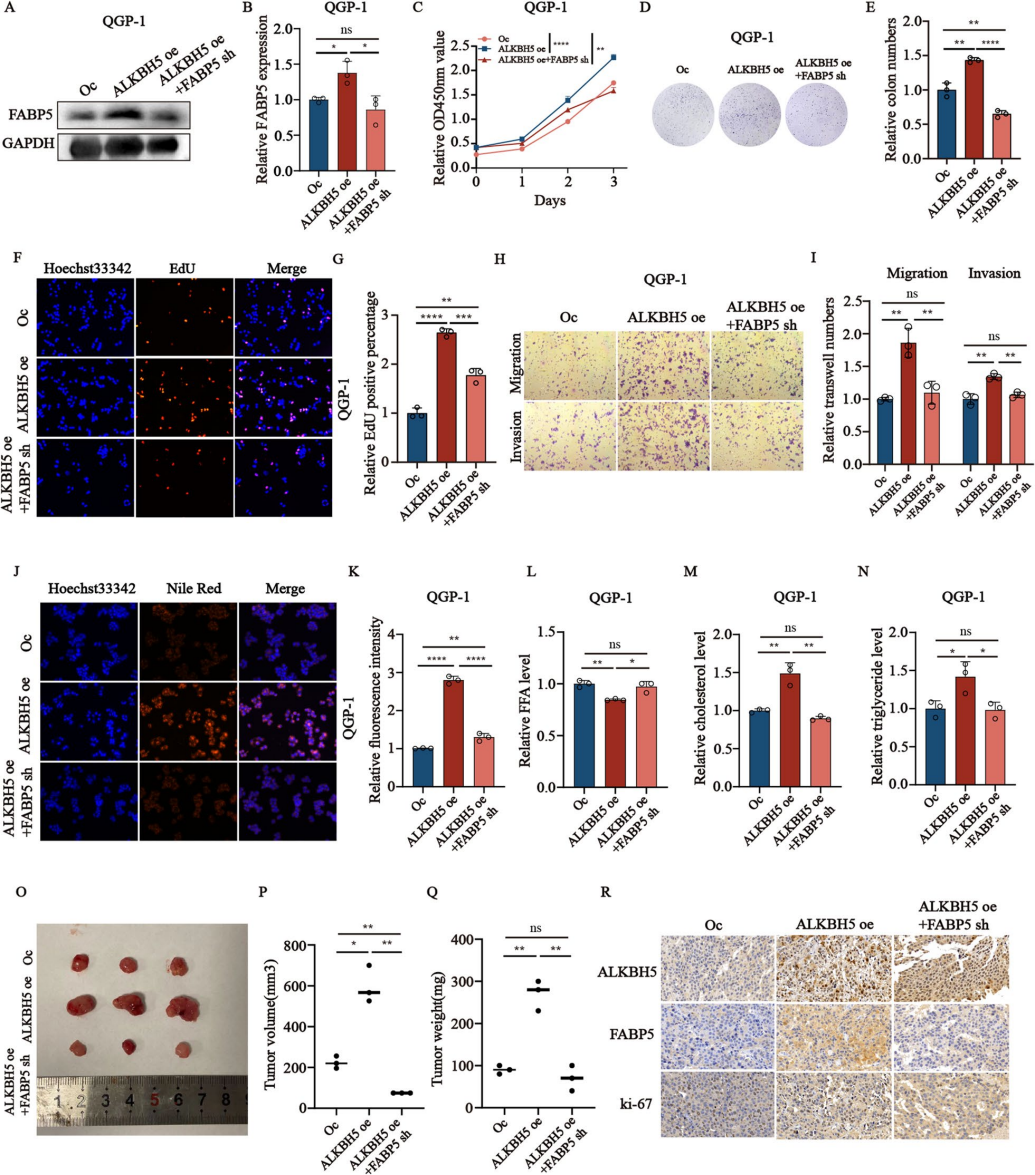
Knockdown of *FABP5* reverses the proliferation, migration, and invasion of cells with *ALKBH5* over-expression. **A** qRT-PCR and **B** Western blotting were performed to examine *FABP5* expression in different transfected cell groups. **C**–**I** Rescue experiments by CCK-8 (**C**), colony formation (**D**, **E**), EdU assays (**F**, **G**), and Transwell assays (**H**, **I**) were conducted to evaluate the effect of *FABP5* interference on the growth and motility of QGP-1 cells with over-expressed *ALKBH5*. **J**–**N** Rescue experiments were performed in QGP-1 cells with over-expressed *ALKBH5* to evaluate the effect of *FABP5* interference on the amounts of LDs (lipid droplets) (**J**, **K**), FFA (free fatty acids) (**L**), total cholesterol level (**M**), and triglyceride (**N**). **O**–**R** Representative subcutaneous xenograft tumor image from the indicated groups (**O**). Tumors were removed 4 weeks after Subcutaneous implantation, followed by volume calculation and weight measurement (**P**, **Q**). Representative IHC staining images of *ALKBH5*, *FABP5*, and *Ki67* (**R**). *p < 0.01, **p < 0.01, ***p < 0.001, ****p < 0.0001

To further examine the role of *FABP5* regulated by *ALKBH5* in cell lines, including the phenotype of proliferation and lipid metabolism, a series of rescue experiments in QGP-1 cells were performed in *ALKBH5* over-expression cell lines with or without *FABP5* knockdown. First, we constructed the stable *ALKBH5* over-expression with *FABP5* knockdown cell lines using lentivirus verified by western blots (Fig. 6A, B). *ALKBH5* over-expression promoted cell proliferation and increased the colony formation of QGP-1 cells, which could be rescued by stable knockdown of *FABP5* (Fig. 6C–E). Rescue with knockdown of *FABP5* also restored the increased DNA replication capacity caused by *ALKBH5* over-expression in QGP-1 cells (Fig. 6F, G). Moreover, *FABP5* inhibition could also partly rescue the ability of migration and invasion in *ALKBH5* over-expression cell lines (Fig. 6H, I). Next, some changes in lipid metabolism were also detected in *ALKBH5* over-expression QGP-1cells with or without *FABP5* knockdown. The results showed that cells over-expressed *ALKBH5* with more accumulation of lipid droplets, less free fatty acids, and higher amounts of cholesterol and triglyceride, which could be rescued by *FABP5* knockdown (Fig. 6J–N). Moreover, in line with in vitro results, *ALKBH5* over-expression led to the higher rate of tumor formation in subcutaneous xenograft models of QGP-1 cells, proved by the higher tumor weight and volume, which could also be rescued by *FABP5* knockdown (Fig. 6O–Q). Moreover, the level of *ALKBH5* and *Ki-67* was also enhanced by *ALKBH5* over-expression and reduced by *FABP5* knockdown again (Fig. 6R). The above results indicated that *ALKBH5*-medicated *FABP5* plays a crucial role in lipid metabolism and tumor development.

### *FABP5* promotes the proliferation, migration, invasion, lipid metabolism of pNENs

**Fig. 7:**
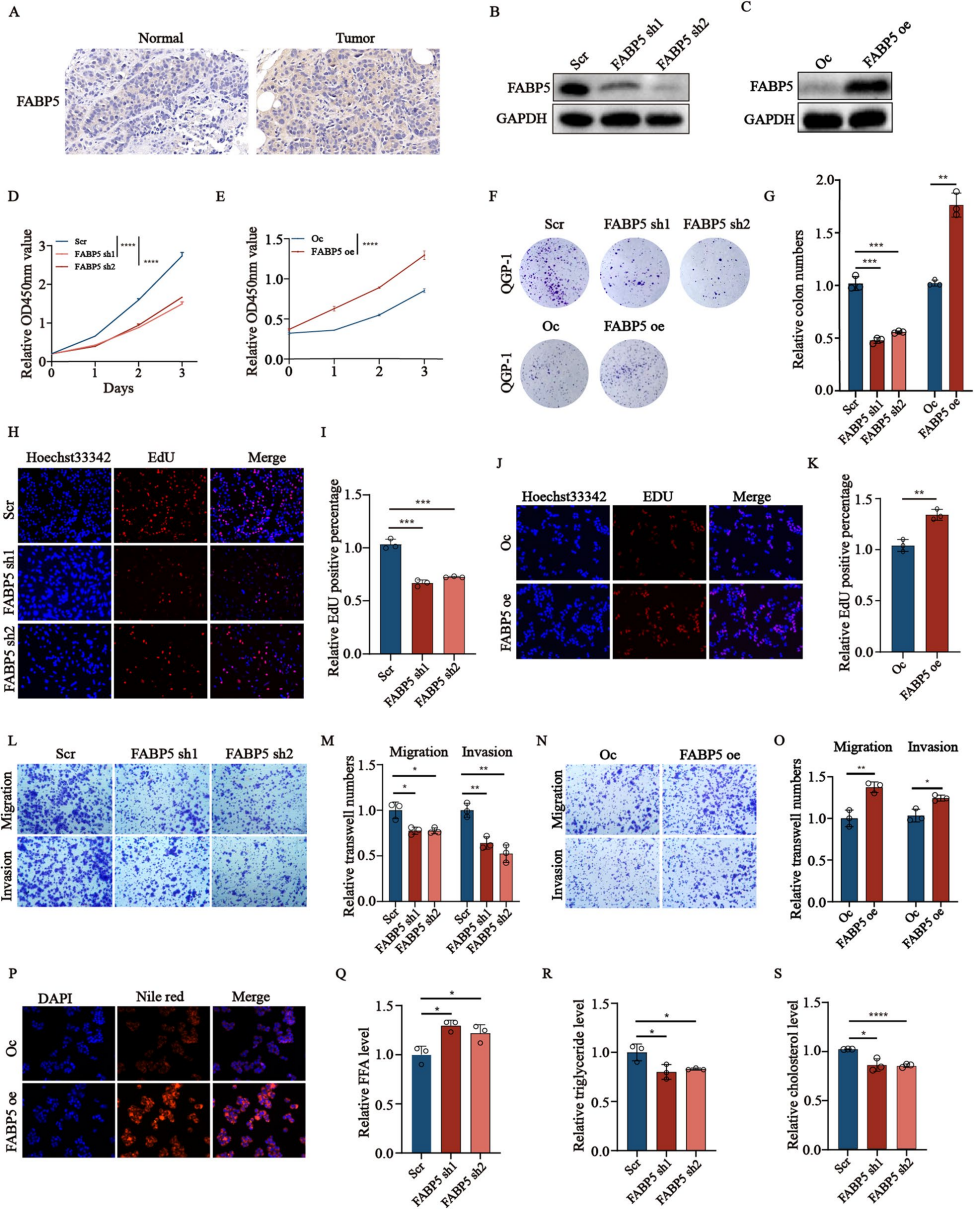
The altered expression of *FABP5* promotes proliferation, migration, and lipid metabolism of QGP-1 cells. **A** Representative images of *FABP5* immunohistochemistry staining in pNENs samples were shown. **B**, **C** The knockdown and overexpression rate of *FABP5* was detected by western blotting. **D**–**J** *FABP5* knockdown inhibited the growth of pNENs, indicated by the results of CCK-8 (**D**, **E**), colony formation (**F**, **G**), and EdU (**H**–**K**) assays in QGP-1 cells with *FABP5* knockdown and overexpression, Edu magnification: ×200. **L**–**O** The transwell analysis was conducted to examine the effect of *FABP5* knockdown on the migration and invasion capabilities of QGP-1 cells, magnification: ×100. **P** LDs (lipid droplets) were detected using Nile red in QGP-1 cells with *FABP5* knockdown and overexpression, along with the results of relative fluorescence intensity, magnification: ×200. **Q**–**S** The relative amounts of free fatty acids, triglycerides, and cholesterol were measured in QGP-1 cells with *FABP5* knockdown. *p < 0.01, **p < 0.01, ***p < 0.001, ****p < 0.0001

To further evaluate the biological function of *FABP5*, we initially examined the expression of *FABP5* in pNENs tissues and pNENs cell lines verified by immunohistochemical staining and immunofluorescence (Fig. 7A, Additional file 1: Figure S2A). Next, we knocked down and over-expressed *FABP5* by lentivirus in pNENs. The protein levels of *FABP5* in QGP-1 cells and PNET were detected by western blotting respectively. (Fig. 7B, C, Additional file 1: Figure S2B-C). Subsequently, CCK8 assay and colon formation were performed to evaluate the proliferation rates of pNENs cells. Silencing *FABP5* inhibited the proliferation of pNENs cells, whereas *FABP5* over-expression increased pNENs cell proliferation (Fig. 7D–G, Additional file 1: Figure S2D–G). EdU assay also showed that the DNA replication capacity of pNENs cells was hampered by *FABP5* knockdown. Conversely, ectopic *FABP5* enhanced DNA replication activity in pNENs cells (Fig. 7H–K, Additional file 1: Figure S2H–K). In addition, we also evaluated the function of *FABP5* in migration and invasion by transwell assay. The results revealed that silencing *FABP5* resulted in a lower migration and invasion capacity evidenced by less stained pNENs cells at the bottom of the chamber. Conversely, over-expression of *FABP5* enhanced the ability of migration and invasion in pNENs cells (Fig. 7L–O, Additional file 1: Figure S2L–O). To further prove the role of *FABP5* in lipid metabolism in pNENs. The Nile red staining showed more lipid droplet accumulation in *FABP5* over-expression cell lines (Fig. 7P, Additional file 1: Figure S2P). Similarly, *FABP5* knockdown could increase the amounts of free fatty acids in QGP-1 cells and PNET cells (Fig. 7Q, Additional file 1: Figure S2Q). Triglyceride and total cholesterol levels were decreased in *FABP5*-knockdown cells (Fig. 7R–S, Additional file 1: Figure S2R–S). The above results revealed *FABP5* may play an indispensable role in lipid metabolism.

### *ALKBH5* causes the accumulation of lipids in pNENs through *PI3K/Akt/mTOR* axis

**Fig. 8:**
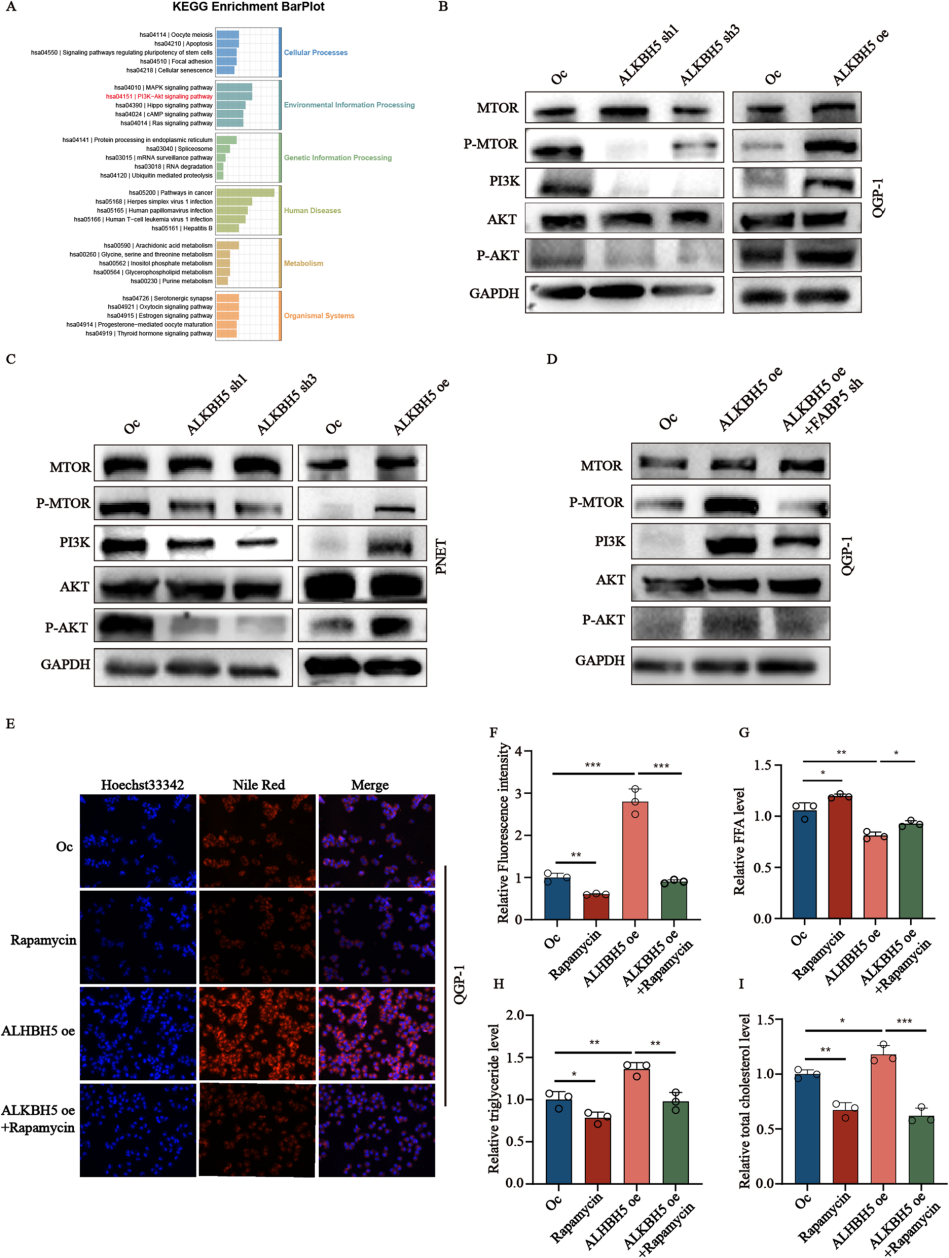
*ALKBH5* regulates lipid metabolism of pNENs by modulating *PI3K/Akt/mTOR* signaling pathway. **A** KEGG enrichment barplot based on *ALKBH5* knockdown and scramble RNA-seq data. **B**, **C** Expression levels of *PI3K/Akt/mTOR* signaling pathway related proteins as determined by western blotting after *ALKBH5* knockdown and over-expression in pNENs cells. **D** Expression levels of *PI3K/Akt/mTOR* signaling pathway related proteins as determined by western blotting in *ALKBH5* over-expression cells with or without *FABP5* knockdown. **E**, **F** LDs (lipid droplets) were detected using Nile red in QGP-1 cells from the indicated groups, along with the results of relative fluorescence intensity, magnification: ×200. **G**–**I** The cellular content of free fatty acids (FFA), triglycerides, and total cholesterol were detected in QGP-1 cells from the indicated groups. *p < 0.01, **p < 0.01, ***p < 0.001

Here, we focus on the *PI3K/Akt/mTOR* signaling pathway through the results of KEEG. To investigate the role of *ALKBH5* in regulating the *PI3K/Akt* signaling pathway in pNENs. We examined the expression of key markers of *PI3K/Akt/mTOR* signaling pathway, the results showed that the expression of *PI3K*, *P-Akt*, and *P-mTOR* were all up-regulated in cells with *ALKBH5* knockdown, whereas the expression of these markers showed the opposite behaviors in cells with *ALKBH5* over-expression (Fig. 8A–C). Moreover, to prove the role of *FABP5* regulated by *ALKBH5* for the *PI3K/Akt/mTOR* signaling pathway. We also examine these markers in cells with *ALKBH5* over-expression and *ALKBH5* over-expression with *FABP5* knockdown. It indicated that *ALKBH5* over-expression could activate the *PI3K/Akt/mTOR* signaling pathway. However, it was also been partly rescued by the inhibition of *FABP5* in *ALKBH5* over-expression cells (Fig. 8D). The above results all showed the *PI3K/Akt/mTOR* signaling pathway was regulated by *ALKBH5* and regulated by *FABP5*. We next examine whether the ability of lipid formation induced by *ALKBH5* could be compromised by Rapamycin, a kind of mTOR inhibitor, the results revealed that this inhibition abrogated the lipid formation ability of QGP-1 cells enhanced by *ALKBH5*. (Fig. 8E–I).

## Discussion

*ALKBH5*, an important component in maintaining the dynamic balance of m6A modification, has become increasingly recognized as a widely up-regulated biomarker in the development of different cancers, including head and neck squamous cell carcinoma [37], colorectal cancer [38], epithelial ovarian cancer [39], pancreatic neuroendocrine neoplasms [40], and is correlated with poor clinical prognosis in patients. However, recent studies also reported the inhibitory role of *ALKBH5* in many cancers, such as gastric cancer [41], and osteosarcoma [42]. Whether *ALKBH5* played a role in cancer promoting or cancer inhibiting may depend on the different target genes regulated by *ALKBH5*. Alternatively, *ALKBH5* is also regulated by different gens in different tumors. The processing of *ALKBH5* functions may involve in the different reading proteins. In addition, the controversial role of *ALKBH5* in different cancers might be the existence of genetic and epigenetic heterogeneities among the cancer cell lines and primary tumor specimens utilized by different research groups. Moreover, the regions of the same or different mRNA transcripts bind by *ALKBH5* is inconsistent and lead to different fates of the target transcripts. Such as, *ALKBH5* promotes the stability of *DDX58* mRNA through *HNRNPC*-mediated RNA stability enhancement due to increased m6A abundance on the 3′-region of *DDX58* mRNA [37]. *ALKBH5*-driven 5ʹ UTR m6A demethylation fine-tunes *SF3B1* translation to impacts genome stability and leukemia progression [43]. Consequently, additional comprehensive investigations are essential to elucidate and resolve these discrepancies. Although there have been reports indicating the upregulation of *ALKBH5* in pNENs [40], the precise role and underlying mechanisms of *ALKBH5* in pNENs remain elusive. Our study also indicated that the expression of *ALKBH5* in pNENs was significantly increased and that m6A modification mediated by *ALKBH5* promoted the survival, proliferation, migration, invasion, and lipid metabolism of pNENs cells.

Lipid accumulation is a driving force in tumor development, as it provides tumor cells with both energy and the building blocks of phospholipids for construction of the cell membranes. Increasing evidence underscores the importance of lipid metabolism in both the initiation and progression of tumorigenesis. Consequently, targeting the process of lipid metabolism for cancer is an optimal strategy for anti-cancer treatment. Current studies have shown that abnormal lipid metabolism may be associated with different types of cancer pathogenesis. Such as, altered lipid metabolism was shown to involve in the progression of glioblastoma [44]. Yin Yang 1(*YY1*) facilitated hepatocellular carcinoma cell lipid metabolism and tumor progression [45]. Moreover, lipid metabolism also plays an indispensable role in drug resistance [46]. For example, Stearoyl-CoA desaturase (*SCD1*) accelerated lipid droplet formation to alleviate chemotherapy-induced ER stress and increase drug resistance in gastric cancer [47]. Interestingly, the altered lipid metabolism was also observed in the tumor micro-environment. Depletion of fatty acid transporter *FATP2* in melanoma cells in an aged micro-environment inhibited lipid accumulation and disrupted their mitochondrial metabolism. In addition, the increased level of cholesterols regulated by cholesterol acyltransferase 1 (*ACAT1*) could enhance CD8^+^ T cell proliferation and support anti-tumor immunotherapy [48]. Interestingly, consistent with the dual role of *ALKBH5* played in tumors, *FABP5* seems also function contradictory effect in different cancers. In colorectal cancer, *FABP5* overexpression exerted an inhibitory influence on cancer progression by reducing lipid accumulation [49]. On the contrary, *FABP5* also play a carcinogenesis role by promoting lipid metabolism in osteosarcoma [50]. This contradictory role may attribute to up-down regulatory mechanisms are different in different cancers, and are also influenced by the interaction of cancer with other organs in the body, thereby warranting further exploration. In the current study, we have uncovered the upregulated *ALKBH5* activated *FABP5* to promote the lipid metabolism of cancer.

Several studies have demonstrated that the intricate interplay between m6A modification and metabolic reprogramming furnished tumor cells with remarkable adaptability to evolving environmental conditions during tumorigenesis. For the connection between m6A modification and glycolysis, m6A-dependent glycolysis enhances the proliferation of colorectal cancer [51, 52]. Elevated *METTL3* expression promoted tumor angiogenesis and glycolysis in gastric cancer. In addition, the *FTO/m6A/PFKP/LDHB* axis is targeted by R-2-hydroxyglutarate, resulting in the suppression of aerobic glycolysis in leukemia. For m6A modification and lipid metabolism, *ACSL4* mediates the function of *METTL5* on fatty acid metabolism and HCC progression. The aberrant m6A modification promotes lipogenesis and contributes to the progression of hepatocellular carcinoma [53]. In addition, the malignant progression of bladder cancer is promoted by m6A-induced lncDBET through *FABP5*-mediated lipid metabolism [31]. In our study, we demonstrated m6A modified *FABP5* plays a crucial role in the progression of pNENs through the altered lipid metabolism. Similarity, a similar study in colorectal cancer has also demonstrated the positive connection between *ALKBH5* and *FABP5* [49]. Nevertheless, the role of other key enzymes involved in m6A functions in the lipid metabolism of other metabolic reprogramming in pNENs are still little known. Moreover, the other epigenetics which not restrict the level of RNA, such as DNA methylation, ubiquitination, phosphorylation, and so on, is still little known in the progression of pNENs, especially for metabolic reprogramming.

*MTOR* signaling pathway, a central signaling pathway controlling tumor metabolism, is one of the signaling pathways that has a fundamental role in the regulation of *PI3K/Akt* and *mTOR* signaling pathway function in gastrointestinal cancer [54]. In colorectal cancer, m6A methylated *EphA2* promotes vasculogenic mimicry via *PI3K/Akt* signaling [55]. Furthermore, *METTL3* promotes the proliferation of retinoblastoma cells by activating *PI3K–Akt–mTOR* signaling pathways [56]. Here, we focus on the *PI3K/Akt/mTOR* signaling pathway through the results of KEEG. The results of western blots have also demonstrated that *ALKBH5* medicated *FABP5* is include in the involvement of *PI3K/Akt/mTOR* signaling. Above all, everolimus, a pharmacological mTOR inhibitor widely used for advanced pNENs patient treatment, the lower expression of *ALKBH5* may further synergize with everolimus to suppress *mTOR* activation and inhibit cancer cell growth.

In summary, the current research explored the biological role and mechanism of *ALKBH5* on pNENs lipid metabolism and malignment behavior. We demonstrated that m6A modification medicated by *ALKBH5* activates the mTOR signaling pathway to promote the lipid metabolism of pNENs through regulating the expression of *FABP5*, thus promoting the malignant progression of pNENs.

### Supplementary Information

**Additional file 1: Table S1.** Primers of genes. **Table S2.** Short hairpin targets. **Table S3.** Antibody information. **Figure S1.** *ALKBH5* over-expression promotes the proliferation, migration, and invasion of PNET. (A) The protein expression of m6A writers (*METTL3, WTAP, METTL14*), erasers (*FTO, ALKBH5*), reader (*YTHDC1*) showed by western blots. (B, C) The efficiency of *ALKBH5* knockdown and over-expression was detected via qRT-PCR and western blot. (D-I) The results of CCK8 (D, E), colony formation (F, G), and EdU assays (H–K) indicated that *ALKBH5* knockdown inhibited the proliferaton of PNET and *ALKBH5* over-expression promoted the proliferation of PNET. (L-O) The results of transwell assay revealed that *ALKBH5* knockdown inhibit the migration and invasion of PNET and *ALKBH5* over-expression had an opposite result, magnification: × 100. **p < 0.01, ***p < 0.001, ****p < 0.0001. **Figure S2.** *FABP5* over-expression promotes the proliferation, migration, and invasion of PNET. (A) The expression of FABP5 in PNET was detected by immunofluorescent imaging. (B, C) The efficiency of *FABP5* knockdown and over-expression in PNET was detected via western blot. (D-G) The results of CCK8 (D, E), colony formation (F, G), and EdU assays (H–K) indicated that *FABP5* over-expression promoted the proliferation of PNET and *FABP5* knockdown had an opposite result. (L-O) The results of transwell assay revealed that *FABP5* over-expression promoted the migration and invasion of pNENs and *FABP5* knockdown had an opposite result, magnification: × 100. (P-S) The relative amounts of free fatty acids, triglycerides, and cholesterol were measured in PNET cells with FABP5 knockdown. *p < 0.01, **p < 0.01, ***p < 0.001, ****p < 0.0001.

## Data Availability

All of the data of this study are available from the corresponding author.

## Abbreviations

pNENs: Pancreatic neuroendocrine neoplasms
m6A: *N*6-Methyladenosine
*ALKBH5*: AlkB homolog 5
*FABP5*: Fatty acid-binding protein 5
MeRIP: Methylated RNA immunoprecipitation
RIP: RNA immunoprecipitation
DAA: 3-Deazaadenosine
CCK8: Cell counting kit-8
EdU: 5-Ethynyl-2ʹ-deoxyuridine
qRT-PCR: Quantitative real-time polymerase chain reaction
GAPDH: Glyceraldehyde-3-phosphate dehydrogenase
