# Supplementary material for: ALKBH5 enhances lipid metabolism reprogramming by increasing stability of FABP5 to promote pancreatic neuroendocrine neoplasms progression in an m6A-IGF2BP2-dependent manner
Source: J Transl Med. 2023 Oct 19;21:741. doi: 10.1186/s12967-023-04578-6 (PMC10588038; doi:10.1186/s12967-023-04578-6)

**Table S1 Primers of genes**

| **Gene names** | **Sequence(5’-3’)** |
| --- | --- |
| FABP5 forward | TGAAGGAGCTAGGAGTGGGAA |
| FABP5 reverse | TGCACCATCTGTAAAGTTGCAG |
| ALKBH5 forward | CGGCGAAGGCTACACTTACG |
| ALKBH5 reverse | CCACCAGCTTTTGGATCACCA |
| GAPDH forward | GGAGCGAGATCCCTCCAAAAT |
| GAPDH reverse  ACTB forward  ACTB reverse  SREBF2 forward  SREBF2 reverse  CD36 forward  CD36 reverse  CPT1A forward  CPT1A reverse  ACSL1 forward  ACSL1 reverse  ACSL3 forward  ACSL3 reverse  SCD1 forward  SCD1 reverse  ACACA forward  ACACA reverse  MLYCD forward  MLYCD reverse  HMGCR forward  HMGCR reverse | GGCTGTTGTCATACTTCTCATGG  CATGTACGTTGCTATCCAGGC 　 　CTCCTTAATGTCACGCACGAT  CCTGGGAGACATCGACGAGAT  TGAATGACCGTTGCACTGAAG  GGCTGTGACCGGAACTGTG  AGGTCTCCAACTGGCATTAGAA  TCCAGTTGGCTTATCGTGGTG  TCCAGAGTCCGATTGATTTTTGC  CCATGAGCTGTTCCGGTATTT  CCGAAGCCCATAAGCGTGTT  GCCGAGTGGATGATAGCTGC  ATGGCTGGACCTCCTAGAGTG  TCTAGCTCCTATACCACCACCA  TCGTCTCCAACTTATCTCCTCC  ATGTCTGGCTTGCACCTAGTA  CCCCAAAGCGAGTAACAAATTCT  ACGTCCGGGAAATGAATGGG  GTAACCCGTTCTAGGTTCAGGA  TGATTGACCTTTCCAGAGCAAG  CTAAAATTGCCATTCCACGAGC |

**Table S2 Short hairpin targets**

| **Gene names** | **Target sequence(5’-3’)** |
| --- | --- |
| FABP5 sh1 | GGCGCCTGGTGGACAGCAAAG |
| FABP5 sh2  ALKBH5 sh1  ALKBH5 sh3  IGF2BP2 sh1  IGF2BP2 sh2 | GCAATGGCCAAGCCAGATTGT  CCACCCAGCTATGCTTCAGAT  CCTCAGGAAGACAAGATTAGA  AGTGAAGCTGGAAGCGCATAT  CAGTGCTGAGATAGAGATTAT |

**Table S3 Antibody information**

| **Antibody** | **Company** | **Catalogue** | **Dilution ratio** |
| --- | --- | --- | --- |
| GAPDH | Proteintech | 60004-1-Ig | 1：5000 |
| mTOR | CST | 2983S | 1：1000 |
| p-mTOR2481 | CST | 2974S | 1：1000 |
| PI3K | Proteintech | 20584-1-AP | 1：1000 |
| AKT | CST | 9272S | 1：1000 |
| p-AKT ser473 | CST | 4060T | 1：1000 |
| FABP5 | CST | 39926T | 1：1000 |
| ALKBH5 | Abcam | ab195377 | 1：1000 |
| Goat Anti-Rabbit IgG | CWBIO | CW0103S | 1：2000 |
| β-Tubulin | Proteintech | 10068-1-AP | 1：5000 |
| IGF2BP2 | Abcam | ab124930 | 1：5000 |

**Figure S1 *ALKBH5* over-expression promotes the proliferation, migration, and invasion of PNET.**


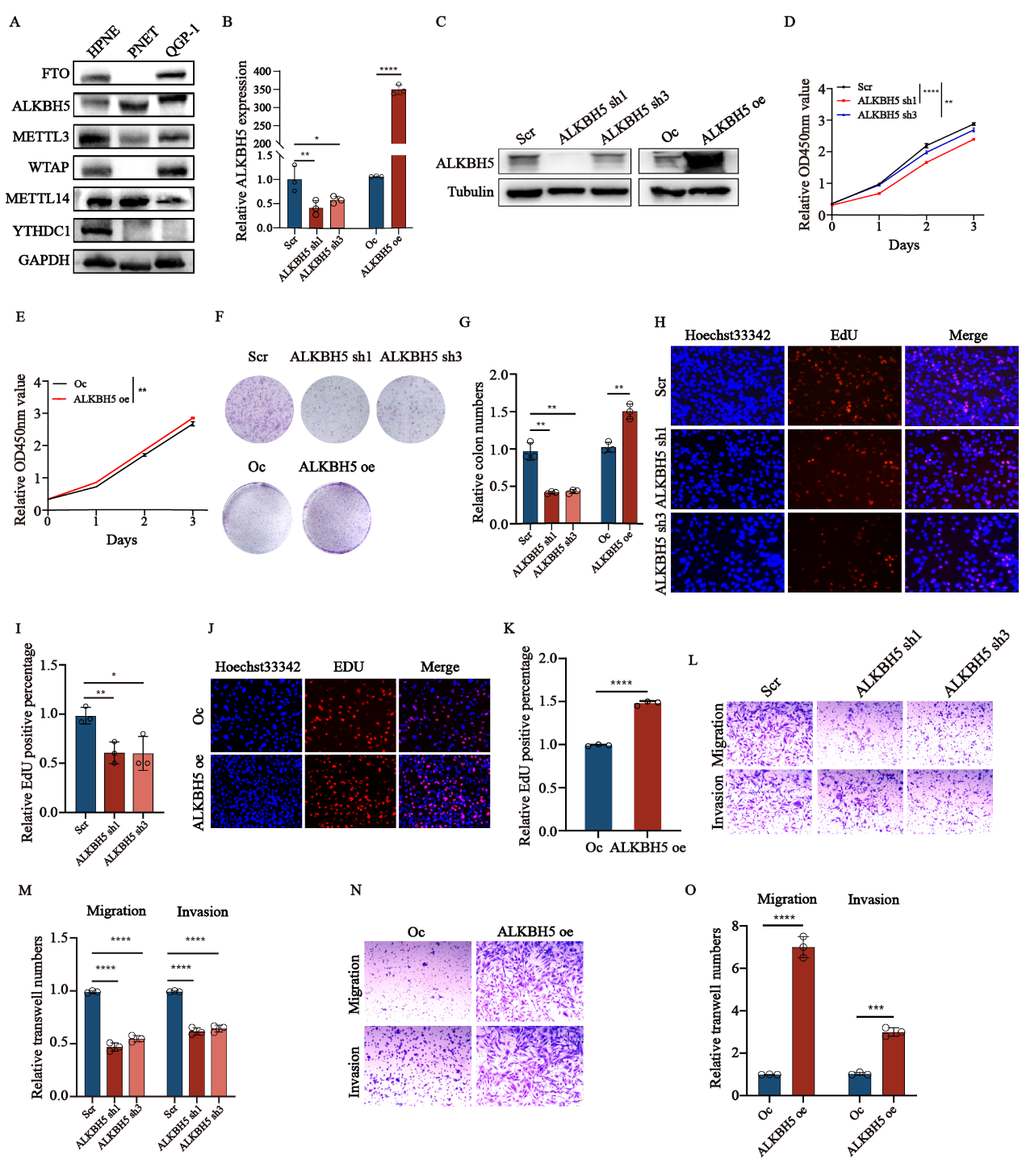


1. The protein expression of m6A writers (*METTL3, WTAP, METTL14*), erasers

(*FTO, ALKBH5*), reader (*YTHDC1*) showed by western blots. (B, C) The efficiency of *ALKBH5* knockdown and over-expression was detected via qRT-PCR and western blot. (D-I) The results of CCK8 (D, E), colony formation (F, G), and EdU assays (H-K) indicated that *ALKBH5* knockdown inhibited the proliferaton of PNET and *ALKBH5* over-expression promoted the proliferation of PNET. (L-O) The results of transwell assay revealed that *ALKBH5* knockdown inhibit the migration and invasion of PNET and *ALKBH5* over-expression had an opposite result, magnification: ×100. **p<0.01, ***p<0.001, ****p<0.0001.

**Figure S2 *FABP5* over-expression promotes the proliferation, migration, and invasion of PNET.**

(A) The expression of FABP5 in PNET was detected by immunofluorescent imaging. (B, C) The efficiency of *FABP5* knockdown and over-expression in PNET was detected via western blot. (D-G) The results of CCK8 (D, E), colony formation (F, G), and EdU assays (H-K) indicated that *FABP5* over-expression promoted the proliferation of PNET and *FABP5* knockdown had an opposite result. (L-O) The results of transwell assay revealed that *FABP5* over-expression promoted the migration and invasion of pNENs and *FABP5* knockdown had an opposite result, magnification: ×100. (P-S) The relative amounts of free fatty acids, triglycerides, and cholesterol were measured in PNET cells with FABP5 knockdown. *p<0.01, **p<0.01, ***p<0.001, ****p<0.0001.


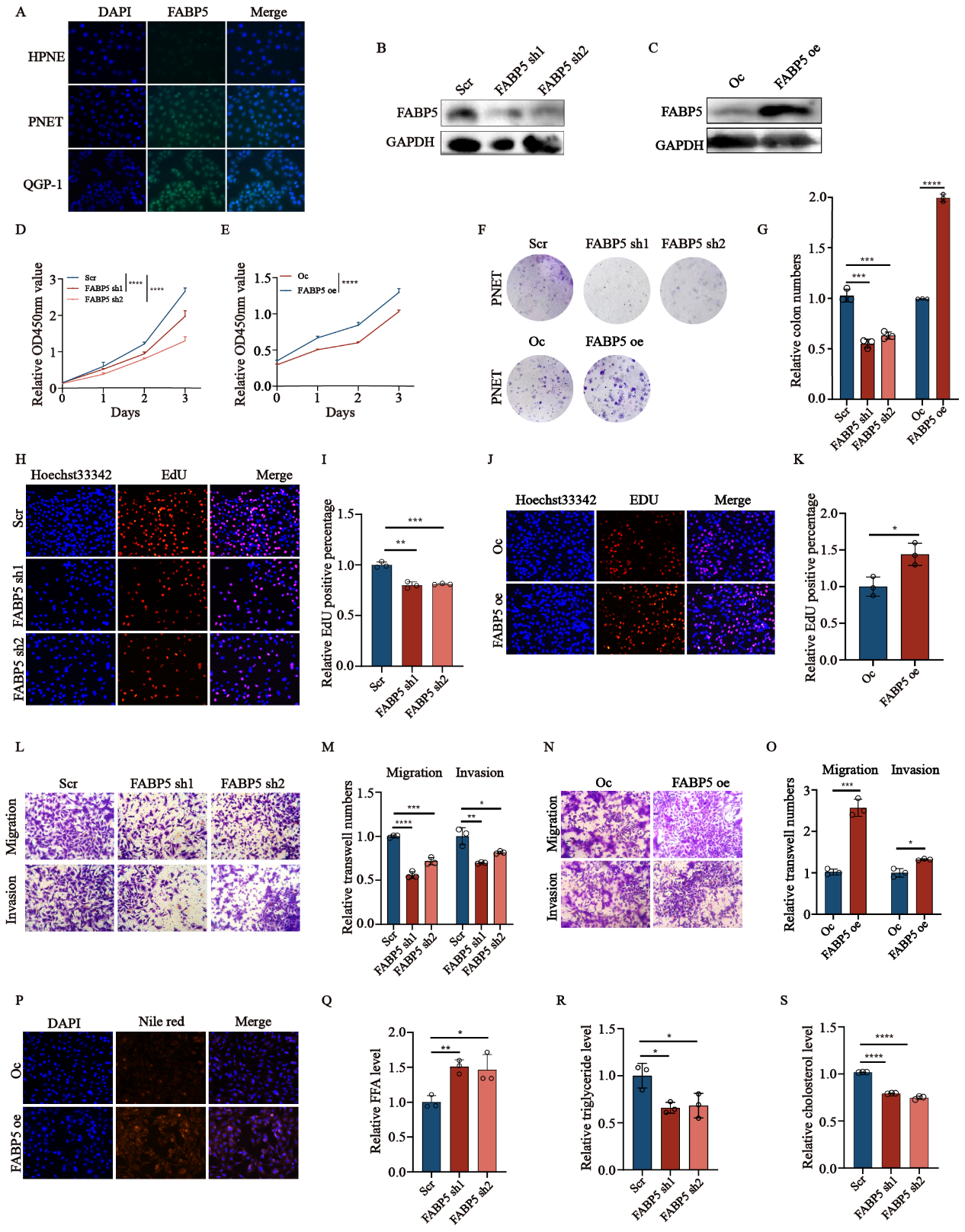

Supplement: Supplementary file 1 — Additional file 1: Table S1. Primers of genes. Table S2. Short hairpin targets. Table S3. Antibody information. Figure S1. ALKBH5 over-expression promotes the proliferation, migration, and invasion of PNET. (A) The protein expression of m6A writers (METTL3, WTAP, METTL14), erasers (FTO, ALKBH5), reader (YTHDC1) showed by western blots. (B, C) The efficiency of ALKBH5 knockdown and over-expression was detected via qRT-PCR and western blot. (D-I) The results of CCK8 (D, E), colony formation (F, G), and EdU assays (H–K) indicated that ALKBH5 knockdown inhibited the proliferaton of PNET and ALKBH5 over-expression promoted the proliferation of PNET. (L-O) The results of transwell assay revealed that ALKBH5 knockdown inhibit the migration and invasion of PNET and ALKBH5 over-expression had an opposite result, magnification: × 100. **p < 0.01, ***p < 0.001, ****p < 0.0001. Figure S2. FABP5 over-expression promotes the proliferation, migration, and invasion of PNET. (A) The expression of FABP5 in PNET was detected by immunofluorescent imaging. (B, C) The efficiency of FABP5 knockdown and over-expression in PNET was detected via western blot. (D-G) The results of CCK8 (D, E), colony formation (F, G), and EdU assays (H–K) indicated that FABP5 over-expression promoted the proliferation of PNET and FABP5 knockdown had an opposite result. (L-O) The results of transwell assay revealed that FABP5 over-expression promoted the migration and invasion of pNENs and FABP5 knockdown had an opposite result, magnification: × 100. (P-S) The relative amounts of free fatty acids, triglycerides, and cholesterol were measured in PNET cells with FABP5 knockdown. *p < 0.01, **p < 0.01, ***p < 0.001, ****p < 0.0001. [file 12967_2023_4578_MOESM1_ESM.docx]
